# Scale-free networks in metabolomics

**DOI:** 10.6026/97320630014140

**Published:** 2018-03-31

**Authors:** Hema Sekhar Reddy Rajula, Matteo Mauri, Vassilios Fanos

**Affiliations:** 1 Neonatal Intensive Care Unit, Department of Surgical Sciences, Neonatal Pathology and Neonatal Section, University of Cagliari, Cagliari, Italy; 2 PhD student Marie Sklodowska-Curie CAPICE Project; 3 University of Cagliari, Cagliari, Italy

**Keywords:** metabolomics, scale-free networks, complex systems, pathways, modelling, metabolites

## Abstract

Metabolomics is an expanding discipline in biology. It is the process of portraying the phenotype of a cell, tissue or species organism using a comprehensive set of metabolites. Therefore, it is of interest to understand complex systems such as metabolomics using a scale-free topology. Genetic networks and the World Wide Web (WWW) are described as networks with complex topology. Several large networks have vertex connectivity that goes beyond a scale-free power-law distribution. It is observed that (a) networks expand constantly by the addition of recent vertices, and (b) recent vertices attach preferentially to sites that are already well connected. Scalefree networks are determined with precision using vital features such as a structure, a disease and a patient. This is pertinent to the understanding of complex systems such as metabolomics. Hence, we describe the relevance of scale-free networks in the understanding of metabolomics in this article.

## Background

Metabolomics is the powerful high throughput technology in light of the whole set of metabolites that provide potential information since it measures and quantifies the outcome of cellular metabolism [1]. A critical challenge for biology and medicine is to achieve a comprehensive understanding of the operation of complex cellular systems as a whole since extensive knowledge about the individual components of living cells is insufficient. The dynamic behavior of biological systems is regulated by complex network of interactions between individual components [2].

**Figure 1:**
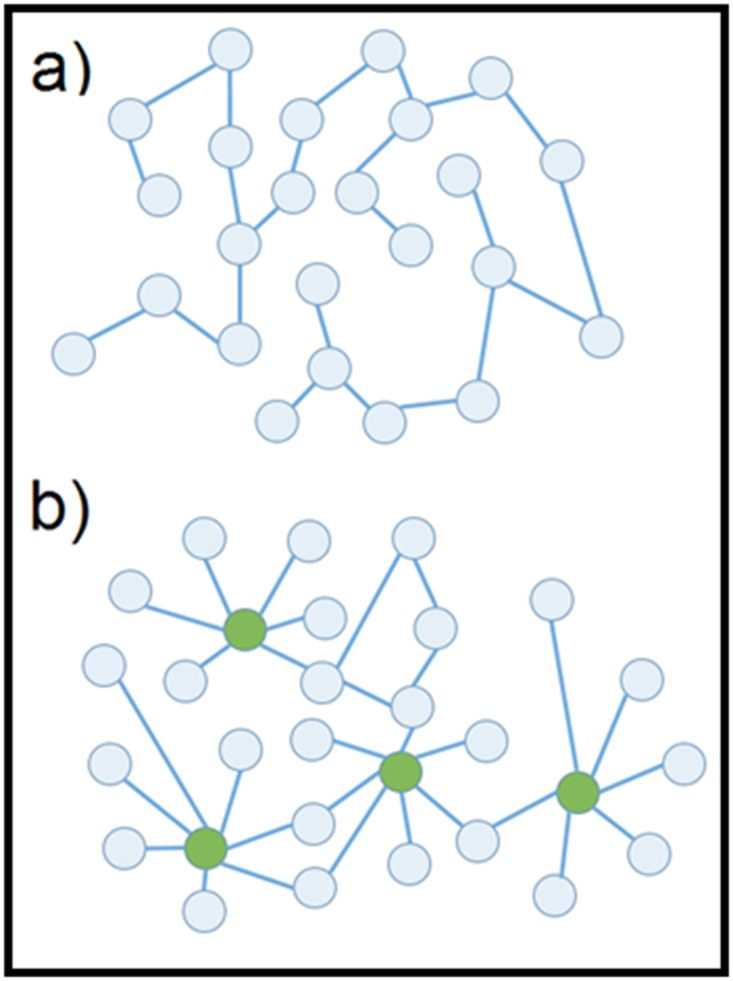
Different networks are shown. (a) Example of a network with the Poisson (Gaussian) typology: the nodes (light blue circles) are identical with few links. (b) Example of a scale-free network: not all the nodes are the same. Some (the green ones) have a huge number of links (hubs) and are much more important than the others. This image is adapted with permission from elsewhere [4].

Complex systems are often portrayed or modeled as networks. The study of complex systems has been carried out traditionally resorting to the mathematical theory of graphs, where each constituent element of the network receives the name of vertex or node, and the connections between them are called edges or links [3] (Figure 1 and Figure 3). The node may be a gene, a protein, or a metabolite. The edge (or link) indicates the interactions of the nodes of a network, for instance, metabolites participation in the same metabolic reactions, or the ligand between protein and protein, or the connections between diseases based on a common genetic origin or shared phenotypical characteristics [4]. Networks grow over time and develop. The hypothesis of preferential attachments ends up in scale-free networks. There is a higher probability of attaching to nodes having the higher number of edges in preferential attachment theory.

Metabolomics deals with essential and vital associations in the scope of scale-free networks. In these interconnecting frameworks, not all the elements are of the same value, some have one or couple of associations though others have numerous such associations. The decade-old disclosure of scale-free networks was one among those events that had helped flip the rise of network science. This is a new research field with its extraordinary arrangement of difficulties and achievements [5]. It should be noted of that the scale-free network systems help to understand metabolism. It also finds application in a number of circumstances such as socio-economic features, intimate relations, stock market associations, organization of hospital (hub and spoke), roads and railway systems. Social networks also illustrate scale-free network systems. Metabolomics will make it conceivable to practice a prospective medicine aimed towards conserving the state of health instead of current reactive medicine that tends to formulate diagnoses and treat diseases already in progress [6].

## Hubs

Small-degree nodes are the foremost abundant in a scale-free network. However, the frequency of high-degree nodes declines relatively slow. Thus, so-called hubs exist in nodes that have degrees in abundant higher than average (Figure 1). Random node disruptions do not result in a significant loss of connectivity due to the heterogeneity of scale-free networks. Nonetheless, the loss of the hubs causes the breakdown of the network into isolated clusters [7].

## Description

### Scale-free networks and complex systems

**Figure 2:**
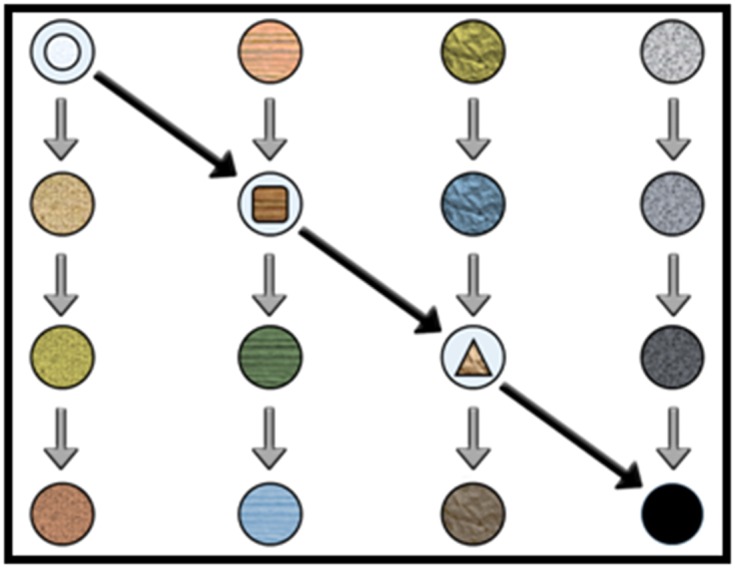
An alternative way to move towards the metabolic processes. Metabolism implies several interconnections between metabolites. The grey arrows represent four individual metabolic pathways as described in biochemistry textbooks. The black arrows, on the opposite hand, show the opportunities offered by metabolomics: there is a metabolic pathway that connects these four pathways through the intermediaries of every pathway. This new approach (highlighted in black) is necessary compared to the traditionally analyzed metabolic pathways. This image is adapted with permission from elsewhere [4].

**Figure 3:**
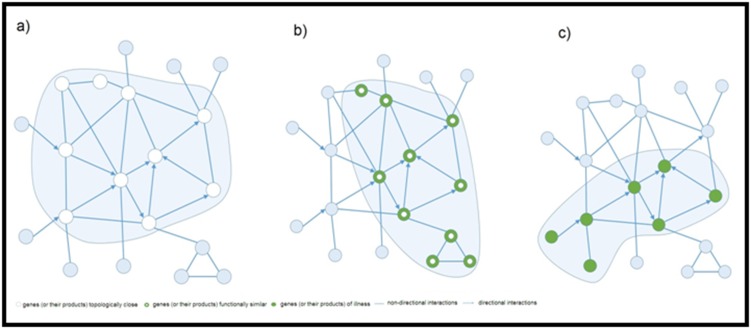
(a) A topological module represents an area of the network densely packed with nodes and links, wherever nodes have a larger tendency to be connected to the nodes of the same area instead of the nodes placed outside the zone itself; (b) A functional module represents the aggregation of nodes with similar or related function within the same zone, wherever the function captures the role of a gene or a product, a protein or a metabolite, within the outline recognizable phenotypes; (c) The illness module represents a set of elements of a network to a cellular function, the destruction of that ends up in a very specific phenotype of the disease. This image is adapted with permission from elsewhere [4].

Scale-free networks share two significant functional characteristics. First, they are differentially sensitive to damage. This implies that if a small, peripheral node stops functioning, the network is very likely to keep working without any issue. By contrast, if a hub is damaged, the functionality of the complete network is likely to be risked [8]. The existence of hubs makes it possible to get information from a part of the network to a different network so as to arrive more quickly and easily (with fewer "jumps") provided they opt for the most effective interconnected route [3]. A good number of well-known complicated systems adopt a scale-free topology with all probabilities without considering structural and design aspects. These include genetic regulatory networks, protein networks, metabolic networks, neural networks, networks of communication and computers (internet, telephone networks, etc.), social networks (friendships, sexual contacts, collaborators scientists and authors of publications, unfolding of diseases (Figure 2 and Figure 3), ecological networks (trophic interactions in an exceeding ecosystem) and several other examples [3]. In this context, an alternative way to describe metabolic processes is shown in Figure 2.

We account for the existence of a high degree of self-organization characterizing the large-scale properties of complex networks. We explore many large databases describing the topology of huge networks that span across numerous fields such as the World Wide Web or citation patterns in science. We show the identity of its constituents independent of the system. The likelihood P(k) is described as a vertex within the network interacting with k different vertices which decays as a power law following P(k) ∼ k -γ, where γ is the degree component [9]. The result shows that massive networks self-organize into a scale-free state. This feature surpasses all existing random network models. We tend to show that existing network models fail to incorporate two key features of real networks namely, growth and preferential attachments in order to describe the origin of this immutable scale. We incline to show that they are responsible for the power-law scaling observed in real networks while describing models incorporating these two ingredients. Thus, we infer that these ingredients play a vital role in the formation of many complex systems [9].

Table 1 shows some examples of systems governed by laws of scale-free networks, which range from our brains to the social networks, from the stock exchange to the writing of scientific articles, from the electricity grid to the telephone network in a large country [4]. It is clear from examples that scale-free networks in biological systems have valuable consequences (facilitate chemical diversity with minimum energy cost, reduce the transition time between metabolic states, decrease the consequences of biochemical or genetic errors, among others) [3].

### Scale-free networks in clinical metabolomics

**Figure 4:**
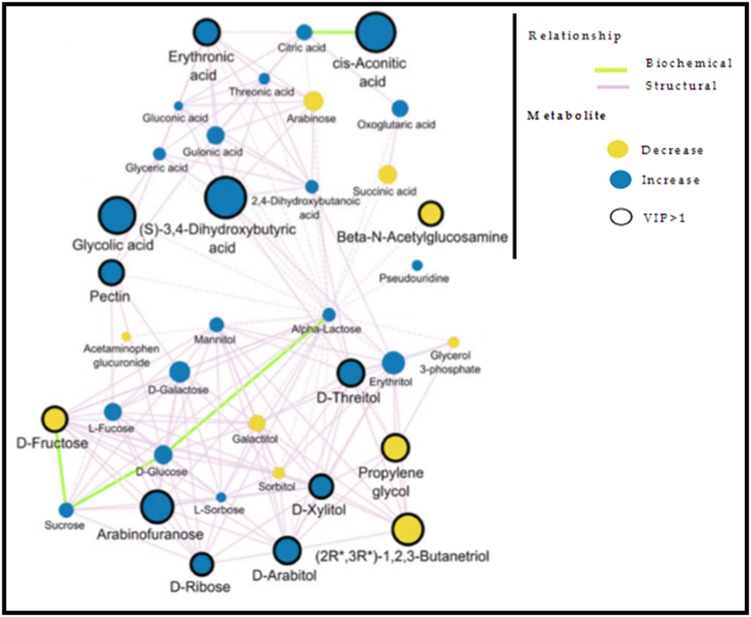
Scale-free networks in the clinical setting: Biochemical and structural similarity network showing changes in urinary metabolites between ASD cohorts and control (detail). Nodes signify metabolites and show the direction of the fold change in ASD versus control and the multivariate importance (VIP) of metabolic changes between ASD cohorts and control. Thick black borders identify metabolites with VIP > 1. The thin-hashed lines indicate maximum structural similarities between metabolites, which didn't meet the structural similarity threshold (solid edges). This image is adapted with permission from elsewhere [10].

A multivariate model has been used to infer the variables of importance for a network model of interaction between metabolites. A network-based approach to ASD description is shown elsewhere [10] as illustrated in Figure 4. A network model of the ASD patient's metabolome using a supervised multivariate model is described to classify the metabolome alterations between autistic spectrum disorders (ASD) patients and controls as well as siblings of autistic patients.

## Conclusion

A complete understanding for the construction of complex systems whose structures adopt scale-free network topologies is important and highly relevant. The hubs of a network (example asthma) assumes that (a) a component that acts as a hub in a process can perform a distinct role in another, and (b) that the importance of hubs could vary throughout the disease and from acute situations to chronic conditions [3]. This strategy is explored in chronic obstructive pulmonary disease (COPD) [11] to define potential therapeutic targets [3]. The near future foresees a change in the methodology for the understanding of diseases. The challenge is to combine big data provided by the genomics, transcriptomics, proteomics and metabolomics with complex systems science, systems biology, and system medicine the body. The limitations in interpreting the nature of interactions with the nature of the objects that interact are realized. The map of connections between elements is vital to the understanding of the requirement to interchange the analytical approach. We need this map to relinquish content to the integral interpretation of available data [3].

Protein-protein interaction and metabolic networks are scale-free where the degree distribution features a power-law. The most noticeable consequence of this property is the presence of a number of extremely connected hubs that hold the full network together.

The biological role and stimulating behaviour of hubs allowed their grouping into "party" hubs (perform inside modules and coordinate particular cellular processes) and "date" hubs (link completely different processes and organize the interactome) [9]. The role of scale-free networks in the understanding of the diseases is predictable in future [8].

## Figures and Tables

**Table 1:** Systems governed by the laws of scale-free networks

Scale-free networks	Nodes (vertex)	Edges (Links)
Scientific research	Scientists	Writing of scientific articles as co-authors.
Cell metabolism	Metabolites	Participation in the same reactions.
Hospital system	Hospitals	Hospital network
World Wide Web	Social networks (Twitter, LinkedIn, Facebook etc.)	URLs (Uniform Resource Locators)
Stock market	The blue chip shares on the market	Stock market trend
Intimate relations	Persons	Intimacy
Brain	Six areas of great importance	The most important parts of the connectome
Highway system	Cities	Highway network
Railroad system	Stations	Railroad network
Hollywood	Actors	Participation in the same movie.
